# Interplay between Endoplasmic Reticulum (ER) Stress and Autophagy Induces Mutant p53H273 Degradation

**DOI:** 10.3390/biom10030392

**Published:** 2020-03-03

**Authors:** Alessia Garufi, Giulia Federici, Maria Saveria Gilardini Montani, Alessandra Crispini, Mara Cirone, Gabriella D’Orazi

**Affiliations:** 1 Department of Research and Advanced Technologies, IRCCS Regina Elena National Cancer Institute, 00144 Rome, Italy; alessiagarufi@yahoo.it (A.G.); giulia.federici@ifo.gov.it (G.F.); 2 University “G. D’Annunzio”, School of Medicine, 66100 Chieti, Italy; 3 Department of Experimental Medicine, Sapienza University of Rome, laboratory affiliated to Istituto Pasteur Italia-Fondazione Cenci Bolognetti, 00161 Rome, Italymara.cirone@uniroma1.it (M.C.); 4 Department of Chemistry and Chemical Technologies, laboratory MAT_IN LAB, Calabria University, 87036 Rende, Italy; alessandra.crispini@unical.it

**Keywords:** p53, mutp53H273, autophagy, endoplasmic reticulum (ER) stress, IRE1α/XBP1, zinc supplementation, 4-PBA, ST-083010, cancer therapy

## Abstract

The unfolded protein response (UPR) is an adaptive response to intrinsic and external stressors, and it is mainly activated by the accumulation of misfolded proteins at the endoplasmic reticulum (ER) lumen producing ER stress. The UPR signaling network is interconnected with autophagy, the proteolytic machinery specifically devoted to clearing misfolded proteins in order to survive bioenergetic stress and/or induce cell death. Oncosuppressor TP53 may undergo inactivation following missense mutations within the DNA-binding domain (DBD), and mutant p53 (mutp53) proteins may acquire a misfolded conformation, often due to the loss of the DBD-bound zinc ion, leading to accumulation of hyperstable mutp53 proteins that correlates with more aggressive tumors, resistance to therapies, and poorer outcomes. We previously showed that zinc supplementation induces mutp53 protein degradation by autophagy. Here, we show that mutp53 (i.e., Arg273) degradation following zinc supplementation is correlated with activation of ER stress and of the IRE1α/XBPI arm of the UPR. ER stress inhibition with chemical chaperone 4-phenyl butyrate (PBA) impaired mutp53 downregulation, which is similar to IRE1α/XBPI specific inhibition, reducing cancer cell death. Knockdown of mutp53 failed to induce UPR/autophagy activation indicating that the effect of zinc on mutp53 folding was likely the key event occurring in ER stress activation. Recently discovered small molecules targeting components of the UPR show promise as a novel anticancer therapeutic intervention. However, our findings showing UPR activation during mutp53 degradation indicate that caution is necessary in the design of therapies that inhibit UPR components.

## 1. Introduction

Tumor suppressor p53 plays a central role in tumor prevention and response to therapies. The presence of a functional p53 pathway is incompatible with neoplastic growth and, for this reason, p53 is the most frequently mutated gene in tumors [1]. The majority of p53 mutations are missense mutations (i.e., R175H, R248Q, and R273H) that are mainly within the DNA-binding domain (DBD), leading to the synthesis of p53 proteins that are unable to bind target gene promoters; however, some mutant (mut) p53 proteins can physically bind other transcription factors, profoundly remodeling the cancer cell transcriptome and proteome [2]. Mouse models of different hotspot mutp53 proteins and clinical data from germline and sporadic cancers have established that some mutp53 proteins not only abolish the wild-type (wt) p53 tumor suppressive function, but can become oncogenic, promoting invasion, metastasis, and chemoresistance [3,4]. Mutp53 proteins may acquire a misfolded and partially denatured conformation, forming hyperstable micro- and macro-aggregates that cannot undergo degradation, with their accumulation in tumors [5]. This aggregation may result from loss or alteration of DBD-bound zinc, which is necessary for the thermodynamic stability of the DBD and is needed for the wtp53 oncosuppressor function [6]. Preventing mutp53 accumulation provides an important chemopreventive and chemotherapeutic anticancer strategy and, in the last few years, many small molecules have been identified to induce mutp53 downregulation and/or reactivation of wtp53 (which is inhibited by mutp53 as a dominant negative effect) [7,8]. Our previous studies showed that zinc supplementation modifies the equilibrium between p53 mutant and wild-type conformation, positively reactivating wtp53 functions of some of the most frequently p53 mutated residues, such as Arg175 and Arg273 [9,10,11,12]. The reactivation of wtp53 conformation results in the re-establishment of canonical DNA binding activity and the transcription of target genes, inducing apoptosis and inhibition of tumor growth, in vitro and in animal models [9,10,11,12]. We also showed that a curcumin-based zinc complex [13] induces mutp53 degradation through autophagy, in part depending on the wtp53-induced target gene DRAM (damage-regulated autophagy modulator) [14,15,16], and in line with the notion that mutp53 blocks autophagy while wtp53 induces it [17].

Macroautophagy (hereafter indicated as autophagy), is a dynamic catabolic process that degrades cytoplasmic components, unfolded proteins, and damaged organelles by enwrapping them into lysosomes assisting cells to cope with stress load [18,19]. Autophagy can promote cell survival or death. The process occurs at both the basal level and in response to stress, and it is linked to the endoplasmic reticulum (ER) stress. ER, the principal intracellular organelle responsible for protein folding, localization, and post-translational modifications, undergoes stress when unfolded proteins accumulate into it due to intracellular and extracellular insults (i.e, glucose deprivation, hypoxia, acidosis, inhibition of protein glycosylation, disturbance of intracellular Ca^2+^ stores, oncogenic mutation, etc.). ER stress triggers the unfolded protein response (UPR) that, besides inducing autophagy, restores ER homeostasis via the reduction of global protein synthesis and the activation of chaperones and degradation processes [20]. UPR is orchestrated by three main sensors, namely inositol-requiring enzyme 1α (IRE1α), activating transcription factor 6 (ATF6), and protein kinase RNA-like ER kinase (PERK), which regulate many signaling pathways (autophagy, apoptosis, antioxidant response, inflammation, etc.), and ultimately dictate cell survival or death decision [21]. The latter occurs when the adaptive UPR response to stress is overwhelmed [22]. Under basal condition, the ER luminal chaperone BiP/GRP78 protein binds to these UPR molecules from the ER lumen and suppresses their basal activity. During ER stress, misfolded proteins accumulate in the ER lumen remove BiP from IRE1α, PERK, and ATF-6, leading to activation of downstream signaling [23]. The IRE1α signaling pathway induces expression of the transcription factor Xbp1s, which increases the expression of ER chaperons and ER mass, stimulates lipid biogenesis, degrades unfolded proteins to enhance the secretory function of ER, and triggers autophagy [24,25]. The activation of PERK can induce the pro-apoptotic function of UPR through the eIF2α-ATF4-CHOP axis, but it can also induce autophagy, together with IRE1α/XBPI and ATF6 arms [26]. UPR and autophagy are, thus, interconnected processes that share common property to promote the adaption of cells to stress [27] and, for this reason, the dysregulation of one of these processes strongly influences the other. Autophagy has been proposed as an innovative target for anticancer therapies, although its manipulation in cancer is still debated [28]. In addition, targeting specific components of the UPR signaling network is also becoming a novel potential anticancer strategy [21]. Therefore, a better understanding of the interplay between UPR and autophagy may help in elucidating the molecular mechanisms that tip the balance towards cell death or survival. Based on these premises, we asked whether ER stress/UPR activation was involved in zinc-induced mutp53 downregulation. 

## 2. Materials and Methods

### 2.1. Cell Culture and Reagents

The human U373MG (expressing R273H p53 mutation) and T98G (expressing M237I p53 mutation) glioblastoma cell lines, and the HT29 (expressing R273H p53 mutation) and RKO (carrying wild-type p53) colon cancer cell lines were maintained in RPM1-1640 (Life Technologies-Invitrogen); the human HCT116 (carrying wild-type p53) colon cancer cell line was maintained in the Dulbecco modified Eagle’s medium (DMEM) (Life Technologies-Invitrogen). All were supplemented with 10% heat-inactivated fetal bovine serum (FBS) (Corning, NY, USA; 35-079) plus L-glutamine and streptomycin (100 µg/mL) (Corning, NY, USA; 30-002), in 5% CO_2_ at 37 °C.

The following reagents were used: A heteroleptic pentacoordinated (bpy-9)Zn(curc, Cl) complex containing a 4,4’-disubstituted-2,2’-bipyridine as the main ligand, and curcumin (curc) and chloride (Cl) as ancillary ligands (Zn(II)-curc) [13], dissolved in DMSO and used at 100 µg/mL, as reported in [12]; the inhibitor of wtp53 transactivation function pifithryn α (PFT-α) (Enzo Life Sciences, Lausen, Switzerland, BML-GR325), dissolved in DMSO and used at 30 µM [15,29]; the ER stress inhibitor 4-Phenylbutyric acid (4-BPA) (Sigma-Aldrich, #P21005) [30], dissolved in filtered sterile water and used at 2.5 mM; the ER stress inducer Tunicamycin (Tn) (Sigma-Aldrich, #T7765), dissolved in DMSO and used at 1 µg/mL; and the inhibitor of XBP1 cleavage STF-083010 (Sigma-Aldrich, #SML0409) (hereafter indicated as STF) [31,32], dissolved in DMSO and used at 60 μM.

### 2.2. Viability Assay and Cell Death/PI Staining

Subconfluent cells were plated in triplicate in six-well plates and, the day after, were treated with Zn (II)-curc (100 µg/mL) for 24 h. Both adherent and floating cells were collected and cell viability was assessed by Trypan blue (Sigma-Aldrich, #72571) exclusion counting blue (dead)/total cells with a Neubauer hemocytometer using light microscopy.

Cell death was detected by cytofluorimetric analysis of propidium iodide (PI)-stained cells, as previously reported [12]. Briefly, both floating and adherent cells were fixed in 80% ethanol and stained in a PBS solution containing PI (62.5 mg/mL; Sigma-Aldrich, #P4864) and RNase A (1.125 mg/mL; Sigma-Aldrich, #R6148). Samples were acquired with a FACScan instrument (Becton Dickinson) and the percentage of cells in the sub-G1 compartment was calculated using ModFit LT software (Becton Dickinson).

### 2.3. Endoplasmic Reticulum (ER) Staining

Subconfluent cells were seeded on coverslips in 35 mm Petri dishes and, the day after, were treated with Zn (II)-curc (100 µg/mL) for 16 h. After treatment, cells were fixed with 3.7% paraformaldehyde (Thermo Fisher Scientific, #50-980-487) for 10 min at room temperature (RT) and stained with ER Staining Kit-Red Fluorescence (Abcam, #ab139482) following the manufacturer’s instructions. Immunofluorescence was visualized by an Olympus BX53 microscope equipped with epifluorescence, and photographs were taken (×40 objective) using a cooled camera device (ProgRes MF). ImageJ (NIH) software [33] (http://imagej.nih.gov) was used to calculate the relative fluorescence from 40× magnification images and normalized to cell size from phase-contrast images. At least 25 cells were analyzed in duplicate for each group at the same exposure time.

### 2.4. RNA Extraction and Semi-Quantitative Reverse Transcription (RT)-PCR Analysis

Cells were harvested in TRIzol Reagent (Invitrogen, #15596026) and total RNA was isolated following the manufacturer’s instructions. The first strand cDNA was synthesized from 2 μg of total RNA with a MuLV reverse transcriptase kit (TermoFisher Scientific, #28025013). Semi-quantitative Reverse-Transcribed (RT)-PCR was carried out by using Hot-Master Taq polymerase (Geneaid Biotech Ltd., New Taipei City, Taiwan, #TQ050) with 2 μL cDNA reaction and genes specific oligonucleotides under the conditions of linear amplification. The primers used for amplification of *Xbp1s* were as follows: *Xbp1s*-for GGAGTTAAGACAGCGCT; *Xbp1s*-rev TGTTCTGGAGGGGTGAC. PCR products were run on a 2% agarose gel and visualized with ethidium bromide. The housekeeping 28S gene, used as the internal standard, was amplified from the same cDNA reaction mixture. Densitometric analysis was applied to quantify mRNA levels compared to control gene expression.

### 2.5. siRNA Interference

Subconfluent cells were plated in 35 mm Petri dishes and, the day after, were transfected with control pSuper and pSuper-p53 (for p53 interference, si-p53) vectors [34] using the LipofectaminePlus reagent (Invitrogen, #11514-015), according to the manufacturer’s instructions. Twenty-four hours after transfection, cells were trypsinized and replated for the indicated experiments.

### 2.6. Western Blotting

Total cell extracts were prepared by incubation in a lysis buffer (50 mM Tris–HCl, pH 7.5, 150 mM NaCl, 5 mM EDTA, 150 mM KCl, 1 mM dithiothreitol, and 1% Nonidet P-40) (all from Sigma-Aldrich) and a mix of protease inhibitors (cOmplete^TM^, Mini Protease Inhibitor Cocktail, Merck, Life Science S.r.l., Milan, Italy, #11836153001). Then, 15–30 μg of protein lysate was subjected to electrophoresis on 9–18% SDS-PAGE gradient gels (Bio-Rad, #456-1095), according to the manufacturer’s instructions. The gels were transferred to a polyvinylidene difluoride (PVDF) membrane (Immobilon-P, Millipore, #IPVH 00010) for 2 h in Tris-glycine buffer. Membranes were blocked in PBS-0.1% Tween 20 solution containing 3% BSA (Sigma-Aldrich) before probing with the following specific primary antibodies: mouse monoclonal anti-p53 (1:1000) (DO-1, (1:100) (Santa Cruz Biotechnology Inc., Heidelberg, Germany; sc-126,); rabbit polyclonal anti-BiP (1:1000) (Cell Signaling, C50B12 #3177); rabbit polyclonal anti-IRE1 alpha (p-Ser724) (1:1000) (Novus Biologicals, #NB100-2323SS); rabbit polyclonal anti-IRE1 alpha (1:1000) (Novus Biologicals, #NB100-2324); rabbit polyclonal anti XBP1 (1:1000) (Novus Biologicals, #NBP1-77681SS); and rabbit polyclonal anti-LC3B (1:1000) (Sigma-Aldrich, #L7543). Mouse monoclonal anti-β-actin (1:10,000) (Sigma Aldrich, #A5441) was used as a protein loading control. Primary antibodies were detected with the following horseradish peroxidase-labeled secondary antibodies: goat polyclonal anti-mouse IgG-horseradish peroxidase (HRP, BioRad.; #172-1011) and anti-rabbit IgG-HRP (BioRad; #172-1019). Enzymatic signals were visualized by chemiluminescence (ECL Detection system, Amersham GE Healthcare, Milan, Italy, #RPN2106), according to the manufacturer’s protocol.

### 2.7. Statistical Analysis

Each experiment was performed at least three times. Results are expressed as values of mean ± standard deviation (S.D.). Statistical significance was determined using Student’s *t*-tests for two sample comparison. Difference was considered statistically significant when the *p*-value was at least <0.05.

## 3. Results

### 3.1. ER Stress Sis Activated by Zn(II)-Curc in mutp53H273 Cancer Cells

**Figure 1:**
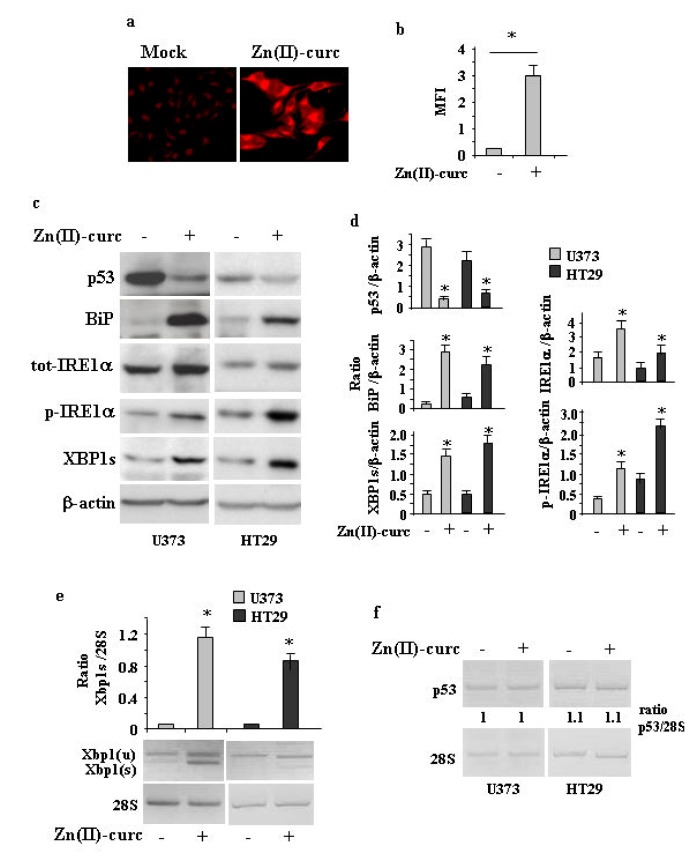
Zn (II)-curc induces endoplasmic reticulum (ER) stress in mutant p53H273-carrying cells. (**a**) Representative photomicrographs of ER-Red Fluorescence staining in U373 cells untreated (Mock) or treated with Zn (II)-curc (100 µg/mL) for 16 h (Original magnification: 40×). (**b**) Quantization of ER content in U373 cells from ER-Red Fluorescence-stained cells. Mean fluorescence intensity (MFI) of each individual cell was normalized to cell size and expressed as fold-change compared with untreated cells at the same time point. Histograms represent the mean ± SD of three independent experiments. * *p* ≤ 0.05. (**c**) Western blot analysis of p53, BiP, total (tot) IRE1α, phosphorylated (p) IRE1α, and XBP1 spliced (s) protein levels evaluated in U373 and HT29 cells untreated or treated with Zn (II)-curc (100 µg/mL) for 24 h. (**d**) Densitometric analysis was performed using Image J software to calculate the ratio of the protein levels, as detected in (**c**), vs. β-actin. Histograms represent the mean ± SD of three independent experiments. * *p* ≤ 0.05. (**e**) Total mRNA was extracted from U373 and HT29 cells untreated or treated with Zn (II)-curc (100 µg/mL) for 24 h. Spliced (s) *Xbp1* gene expression was assayed by the polymerase chain reaction (PCR) of reverse-transcribed cDNA. Densitometric analysis was performed using Image J software to calculate the *Xbp1s*/28S ratio. Histograms represent the mean ± SD of three independent experiments. * *p* ≤ 0.05. (**f**) p53 gene expression was assayed by PCR as in (**e**). The p53/28S ratio is indicated.

As Zn (II)-curc may induce mutp53 protein degradation through autophagy [14,15], and autophagy can be induced by UPR activation [27], we investigated the effect of Zn(II)-curc on ER stress/UPR activation. To address this issue, we used several approaches. U373 cells were treated with 100 μM Zn (II)-curc, the dose that induces mutp53 degradation and autophagy [14,15]. ER analysis by specific fluorescent red labelling shows that Zn (II)-curc caused a marked increase in ER size (Figure 1a,b). This increase correlated with the augmented expression of BiP/Grp78 as well as with the IRE1α and p-IRE1α protein levels in both U373 and HT29 cells (Figure 1c,d), while the other arms of the UPR were not affected in this experimental condition (not shown). Western blot and semiquantitative RT-PCR evidenced spliced (s) *Xbp1* mRNA (Figure 1c–e), in agreement with the notion that activated IRE1α functions as an endoribonuclease, splicing a 26 base pair intron from *Xbp1* mRNA [35]. The Zn (II)-curc-induced activation of IRE1α correlated with the reduction of mutp53 expression levels (Figure 1c), while the p53 gene expression was not affected (Figure 1f). Interestingly, the Zn (II)-curc treatment of T98 cells (expressing M237I p53 mutation), which we previously reported not been affected by Zn (II)-curc at a biological level [12], did not increase BiP levels or reduced p53 protein levels (Supplementary Material Figure S1).

**Figure 2:**
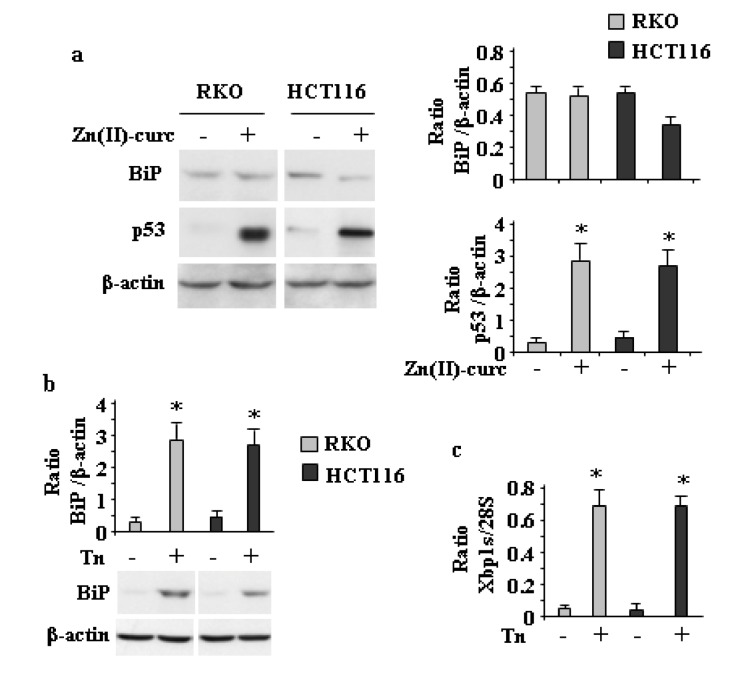
Zn (II)-curc does not affect ER stress in wtp53-carrying cells. (**a**) (left panels) Western blot analysis of BiP and p53 protein levels in RKO and HCT116 cells treated with Zn(II)-curc (100 µg/mL) for 24 h. Densitometric analysis (right panels) was performed using Image J software to calculate the ratio of BiP and p53 protein levels vs. β-actin. Histograms represent the mean ± SD of three independent experiments. * *p* ≤ 0.05. (**b**) Western blot analysis of BiP protein levels in RKO and HCT116 cells treated with Tunicamycin (Tn) (1 µg/mL) for 4 h. (upper panel) Densitometric analysis was performed using Image J software to calculate the ratio of BiP protein levels vs. β-actin. Histograms represent the mean ± SD of three independent experiments. * *p* ≤ 0.05. (**c**) Total mRNA was extracted from RKO and HCT116 cells treated with Tunicamycin (Tn) for 24 h, and *Xbp1* gene expression was assayed by the PCR of reverse-transcribed cDNA. Densitometric analysis was performed using Image J software to calculate the *Xbp1s*/28S ratio. Histograms represent the mean ± SD of three independent experiments. * *p* ≤ 0.05.

To further address the correlation between mutp53H273 degradation and ER stress activation, wtp53-expressing cells were treated with Zn (II)-curc. As shown in Figure 2a, Zn(II)-curc did not increase BiP expression levels or induce *Xbp1* splicing (not shown) in wtp53-expressing cells, while it stabilized endogenous wtp53 protein levels in accordance with previous studies where zinc supplementation induced wtp53 oncosuppressor activities and the transcription of target genes such as p21, Puma, and Bax [14,36]. On the contrary, both BiP (Figure 2b) and *Xbp1* splicing (Figure 2c) were efficiently induced in wtp53-expressing cells by Tunicamycin (Tn), a drug causing ER stress by inhibiting N-linked glycosylation [37]. Altogether, these results indicate that the IRE1α/XBP1 arm of UPR was activated in response to Zn (II)-curc only in mutp53H273 cells.

### 3.2. ER Stress/IRE1α Inhibition Impairs Zn(II)-Curc-Induced Autophagy and Mutp53 Degradation

**Figure 3:**
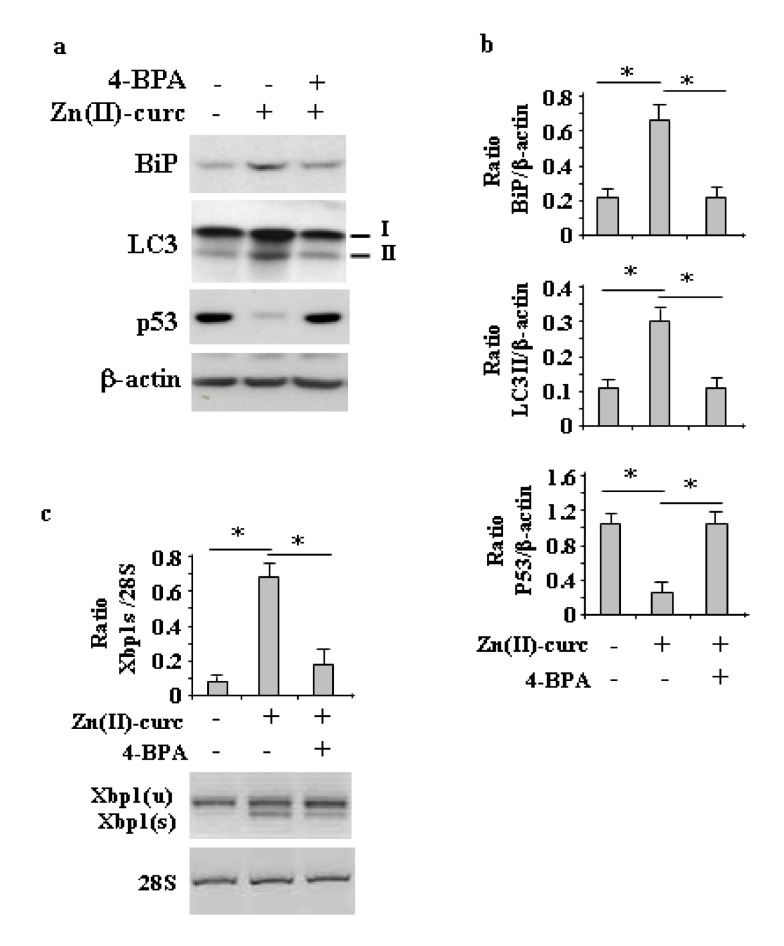
ER stress inhibition impairs Zn (II)-curc-induced autophagy and mutp53 degradation. (**a**) Western blot analysis of BiP, LC3I/II, and p53 protein levels in U373 cells untreated or treated with Zn(II)-curc (100 µg/mL) for 24 h, with or without 1 h pre-treatment with 4-BPA (2.5 mM). (**b**) Densitometric analysis was performed using Image J software to calculate the ratio of the protein levels, as detected in (**a**), vs. β-actin. Histograms represent the mean ± SD of three independent experiments. * *p* ≤ 0.05. (**c**) Total mRNA was extracted from U373 cells untreated or treated as in (**a**). Spliced (s) and unspliced (u) *Xbp1* gene expression were assayed by the PCR of reverse-transcribed cDNA. Densitometric analysis was performed using Image J software to calculate the *Xbp1s*/28S ratio. Histograms represent the mean ± SD of three independent experiments. * *p* ≤ 0.05.

To evaluate the role of ER stress/UPR activation in Zn (II)-curc-induced autophagy, we analyzed the LC3I/II level (a cellular readout of autophagy) [18] by Western blot in U373 cells treated with Zn (II)-curc with or without pre-treatment with 4-Phenyl butyric acid (PBA), a molecular chaperone that dampens the ER stress [30]. The results show reduction of LC3II levels in the presence of 4-BPA, concomitantly to the reduction of Zn (II)-curc-induced BiP protein levels (Figure 3a,b) and *Xbp1* splicing (Figure 3c), suggesting that a possible autophagy inhibition was occurring following ER stress inhibition, which is in agreement with the known role of *Xbp1* splicing in triggering autophagy [38]. Concomitantly, we verified the effect of ER stress inhibition on mutp53 levels and found that mutp53 protein was no longer downregulated in the presence of 4-BPA (Figure 3a,b).

**Figure 4:**
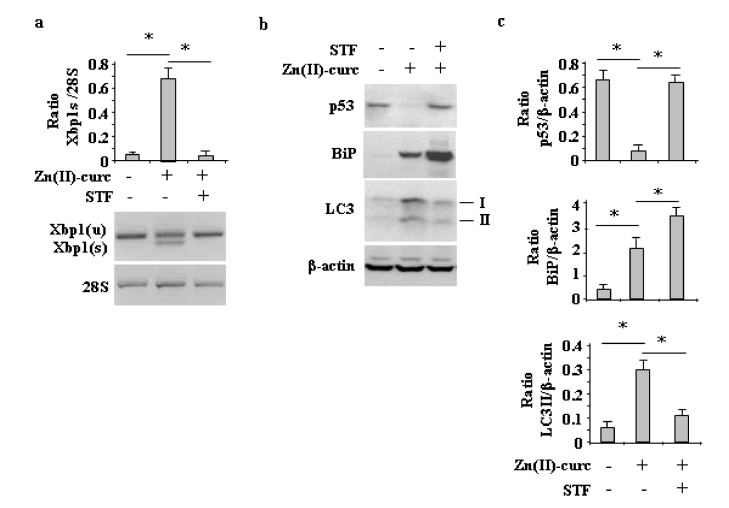
IRE1α inhibition impairs Zn (II)-curc-induced autophagy and mutp53 degradation. (**a**) Total mRNA was extracted from U373 cells untreated or treated with Zn(II)-curc (100 µg/mL) for 24 h, with or without inhibitor of XBP1 cleavage STF-083010 (STF) (60 μM). Spliced (s) and unspliced (u) *Xbp1* gene expression was assayed by the PCR of reverse-transcribed cDNA. (lower panel) Densitometric analysis was performed using Image J software to calculate the *Xbp1s*/28S ratio. Histograms represent the mean ± SD of three independent experiments. * *p* ≤ 0.05. (**b**) Western blot analysis of p53, BiP, and LC3I/II protein levels in U373 cells untreated or treated, as in (**a**). (**c**) Densitometric analysis was performed using Image J software to calculate the ratio of the protein levels, as detected in (**b**), vs. β-actin. Histograms represent the mean ± SD of three independent experiments. * *p* ≤ 0.05.

Similarly, the inhibition of IRE1α-Xbp1 by the small molecule STF [31,32], which indeed counteracted the Zn (II)-curc-induced *Xbp1* splicing (Figure 4a) and slightly increased BiP levels (Figure 4b,c), impaired the Zn (II)-curc-induced increase of LC3II levels and counteracted the Zn (II)-curc-induced mutp53 downregulation (Figure 4b,c). Altogether, these results suggest that the Zn (II)-curc-induced IRE1α-Xbp1 arm of the UPR was able to promote autophagy and mutp53 downregulation.

### 3.3. ER Stress or IRE1α Inhibition Reduces Zn(II)-Curc-Induced Cell Death

**Figure 5:**
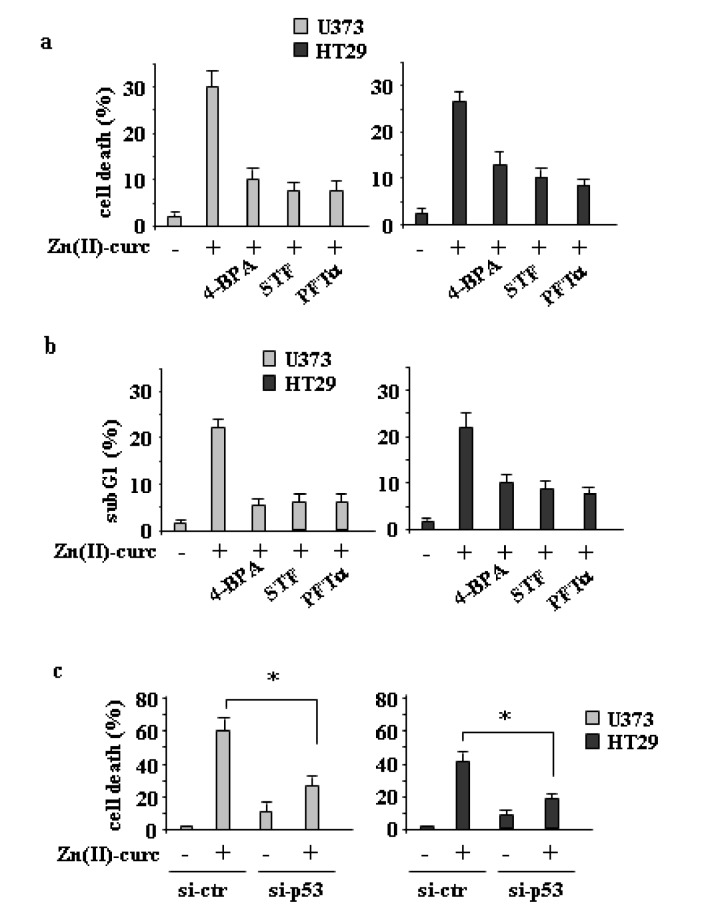
ER stress or IRE1α inhibition impair Zn (II)-curc-induced cell death. (**a**) Cell viability was measured by trypan blue exclusion assay in U373 and HT29 cells untreated or treated with Zn (II)-curc (100 µg/mL) for 24 h, with or without the inhibitor of XBP1 cleavage STF (60 μM), p53 inhibitor PTF-α (30 μM), or 1 h pre-treatment with 4-BPA (2.5 mM), and expressed as percentage ± SD of three independent experiments. * *p* ≤ 0.05 (combination treatments vs. Zn(II)-curc). (**b**) Cytofluorimetric analysis of the SubG1 peak evaluated by Propidium Iodide (PI) staining of U373 and HT29 cells untreated or treated as in (**a**) for 24 h. Percentage of apoptotic cells is shown ± SD of two independent experiments. (**c**) U373 and HT29 cells were transfected with control pSuper (si-ctr) and pSuper-p53 (si-p53) vectors for p53 knockdown and 36 h after transfection, cells were treated with Zn(II)-curc (100 µg/mL) for 24 h. Cell viability was measured by a trypan blue exclusion assay and expressed as percentage ± SD of three independent experiments. * *p* ≤ 0.05.

Next, we evaluated the role of ER stress/IRE1α activation on Zn (II)-curc-induced cell death. To this aim, we combined Zn (II)-curc with either ER stress or IRE1α inhibitors. The results show that both 4-BPA and STF efficiently reduced the Zn (II)-curc-induced cell death (Figure 5a,b), suggesting that the activation of ER stress balanced the cell death/survival equilibrium towards cell death. Similarly, the inhibition of wtp53 transcriptional activity with PFT-α (Figure 5a,b), as well as p53 knockdown (Figure 5c), reduced the Zn (II)-curc-triggered cell death, in agreement with the effect of Zn(II)-curc on the reactivation of wtp53 oncosuppressor functions, as previously reported [12,14,15]. Altogether, these results suggest that both ER stress/UPR activation and wtp53 reactivation by Zn (II)-curc promoted cell death in mutp53 cells.

### 3.4. Mutp53 Knockdown Abrogates the Zn(II)-Curc-Induced ER Stress

**Figure 6:**
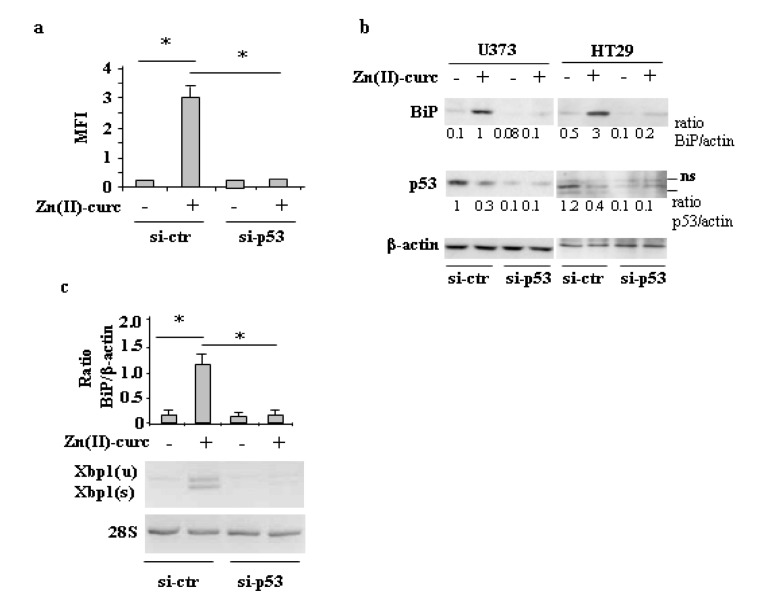
Mutp53 knockdown abrogates the Zn (II)-curc-induced ER stress. (**a**) U373 cells were transfected with control pSuper (si-ctr) and pSuper-p53 (si-p53) vectors for p53 knockdown and 36 h after transfection, cells were treated with Zn (II)-curc (100 µg/mL) for 16 h, before undergoing ER-Red Fluorescence staining. Quantization of ER content in U373 cells from ER-Red Fluorescence-stained cells as evaluated by the mean fluorescence intensity (MFI) of each individual cell normalized to cell size and expressed as fold-change compared with untreated cells at the same time point. Histograms represent the mean ± SD of three independent experiments. * *p* ≤ 0.05. (**b**) Western blot analysis of BiP and p53 protein levels in U373 and HT29 cells transfected for 36 h with si-ctr and si-p53, and then treated with Zn (II)-curc (100 µg/mL) for 24 h. Densitometric analysis was performed using Image J software to calculate the ratio of BiP and p53 protein levels vs. β-actin, as indicated ns: not specific signal. (**c**) Total mRNA was extracted from U373 cells treated as in (**b**), and spliced (s) and unspliced (u) *Xbp1* gene expression were assayed by the PCR of reverse-transcribed cDNA. (upper panel) Densitometric analysis was performed using Image J software to calculate the *Xbp1s*/28S ratio. Histograms represent the mean ± SD of three independent experiments. * *p* ≤ 0.05.

Finally, we asked whether Zn (II)-curc-induced mutp53 downregulation was upstream of the ER stress or rather the consequence of UPR/autophagy activation. To address this issue, p53 was knocked-down with siRNA, and ER stress was analyzed by several approaches. Analysis of ER by immunofluorescence shows that the increase in ER size by Zn (II)-curc was abrogated in the U373 cells undergoing p53 knockdown (Figure 6a); in agreement, the Zn (II)-curc-induced BiP protein levels (Figure 6b) and *Xbp1* splicing (Figure 6c) were strongly reduced by p53 knockdown in both U373 and HT29 cells, suggesting that mutp53H273 degradation by Zn (II)-curc was likely upstream of the ER stress/UPR activation.

## 4. Discussion

**Figure 7:**
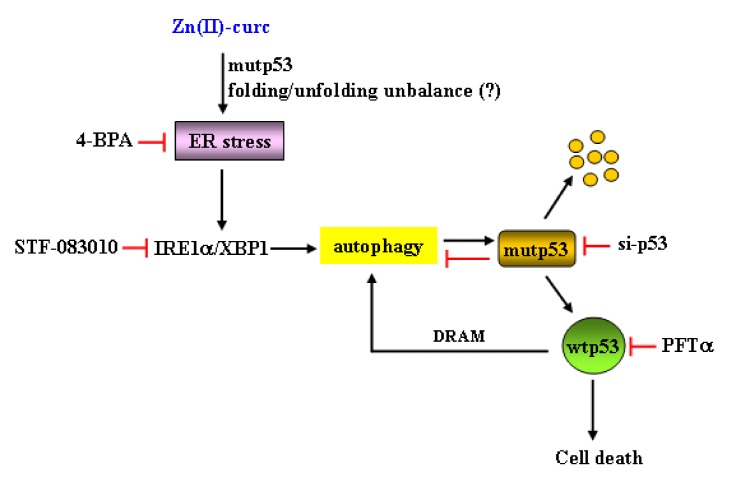
Schematic representation of the interplay between ER stress/unfolded protein response (UPR) activation and autophagy in response to Zn (II)-curc in mutp53H273-carrying cancer cells. Diagram illustration describes the pathways contributing to mutp53H273 degradation by autophagy and the role of ER stress/UPR activation. The arrows indicate activation, and the red lines indicate blocking. The possible starting even could be the Zn-induced mutp53 folding/unfolding balance, as previously reported [8,11,36,37].

Cancer cells are often exposed to intrinsic and external factors that alter protein homeostasis. The consequence is the activation of ER stress and UPR, which is the adaptive mechanism used to cope with ER stress and to restore ER proteostasis [21]. Activation of the UPR through three different but interconnected signaling pathways may favor pro-death or prosurvival signaling and is strictly linked to autophagy. Autophagy is usually a prosurvival mechanism, playing a crucial role in drug resistance, although it may also induce cell death [39,40]. The interplay between autophagy and UPR and their abnormal activation may pave the way to degenerative and chronic diseases including cancer [28,41]. Therefore, understanding the crosstalk between UPR activities and autophagy should help in developing new treatment options for various pathologies, including cancer. Here, we found that autophagy-mediated mutp53H273 degradation following zinc supplementation correlated with activation of ER stress and of the IRE1α/XBPI arm of the UPR. ER stress or IRE1α/XBPI inhibition impaired mutp53 degradation and reduced cell death, suggesting that both ER stress/UPR activation and the clearance of mutp53 by zinc played a pro-death role in mutp53-carrying cells, as summarized in Figure 7.

Mutp53 proteins may acquire a misfolded conformation forming hyperstable micro- and macro-aggregates that cannot undergo degradation, with consequent accumulation in tumors [5]. This aggregation may result from the loss or alteration of DBD-bound zinc, which is necessary for the thermodynamic stability of the DBD, and is needed for wtp53 oncosuppressor function [6]. Our previous studies demonstrated that zinc supplementation was able to modify the equilibrium between the p53 mutant and wild-type conformation, positively reactivating wtp53 functions of some of the most frequently p53 mutated residues, such as Arg175 and Arg273 [9,10,11,12,14,42,43], which is in line with the findings that zinc metallochaperone rescues mutant p53 conformation [44]. Based on these findings, here we can speculate that the effect of zinc on mutp53 folding/unfolding unbalance could act as a cellular stress to trigger UPR activation. Thus, the depletion of mutp53 failed to recapitulate the effect of zinc on UPR activation, and zinc did not activate UPR in wtp53-carrying cells. In addition, zinc treatment of cells carrying a different mutant p53 protein (i.e, M237I) did not induce mutp53 degradation or trigger UPR activation. Therefore, we can reasonably affirm that the UPR activation by zinc is linked to the degradation of the mutp53 protein and, in particular, of mutp53H273. However, further studies are needed to unveil the mechanisms that are involved in UPR activation by degradation of some mutp53 proteins by zinc.

UPR activation is commonly observed in various tumor specimens and correlates with drug resistance [45]. UPR is orchestrated by three main sensors, and our data show that the IRE1α-XBP1 arm of UPR was mainly activated during mutp53 degradation through autophagy, likely in an autoregulatory loop, although the exact mechanism by which mutp53 degradation increases ER stress-induced activation of IRE1α/XBPI remains to be defined. The IRE1α-XBP1 pathway is the most conserved signaling branch of the UPR and plays important roles in both physiological and pathological settings, with its activity having profound effects on disease progression and prognosis [35]. The IRE1α signaling pathway induces the expression of the transcription factor Xbp1s, which increases the expression of ER chaperones and ER mass, stimulates lipid biogenesis, and degrades unfolded proteins to enhance the secretory function of ER and triggers autophagy initiation, mainly serving as a pro-survival pathway in multiple human cancers [35]. XBP1 pathway activation has been shown to induce triple-negative breast cancer progression and is correlated with poor patient survival, suggesting that UPR inhibitors in combination with chemotherapy may improve tumor regression [46]. In addition, unspliced XBP1 is associated with longer survival of breast cancer patients treated with tamoxifen, which is opposite to XBP1 splicing that is associated with shorter survival [47]. Therefore, developing combination therapies that target specific UPR signaling pathways will hopefully bypass anticancer drug resistance. On the other hand, we found here that XBP1 inhibition impaired zinc-induced autophagy and mutp53 degradation, indicating that UPR modulation may achieve different effects depending on cell context. To make the story more complex, mup53 has been shown to favor cancer cell survival and promote cancer progression by its crosstalk with cellular stress pathways [48]. For instance, the downregulation of mutp53 interferes with the downstream activation of the cross-talk between NRF2 and p62, restoring the cytotoxic effect of chemotherapies [49], and mutp53 inhibits the pro-apoptotic and pro-survival UPR effectors PERK and ATF6 in cancer cell lines [50], suggesting that UPR inhibitors combined with mutp53 inhibitors may increase cell death.

## 5. Conclusions

In conclusion, this study demonstrates for the first time that a curcumin-based zinc complex was able to induce UPR activation to trigger autophagy and mutp53H273 degradation, highlighting the interplay between ER stress and autophagy. As UPR activation has both pro-survival and pro-death effects, caution is necessary in the design of therapies that target UPR components in combination with chemotherapies to increase the antitumor response, as they could hamper mutp53 degradation.

## Supplementary Materials

The following are available online at https://www.mdpi.com/2218-273X/10/3/392/s1.
