# Supplementary material for: Interplay between Endoplasmic Reticulum (ER) Stress and Autophagy Induces Mutant p53H273 Degradation
Source: Biomolecules. 2020 Mar 3;10(3):392. doi: 10.3390/biom10030392 (PMC7175121; doi:10.3390/biom10030392)
Supplement: Supplementary file 1 [file biomolecules-10-00392-s001.pdf]

Interplay between Endoplasmic Reticulum (ER) Stress and Autophagy Induces Mutant p53H273 Degradation

Alessia Garufi [1,2], Giulia Federici [1], Maria Saveria Gilardini Montani [3], Alessandra Crispini [4], Mara Cirone [3] and Gabriella D'Orazi [1,*]

Figure 1C, U373
actin

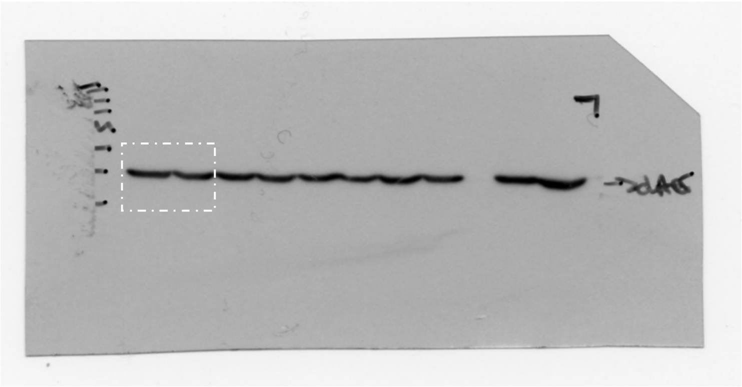

Figure 1C, HT29
actin

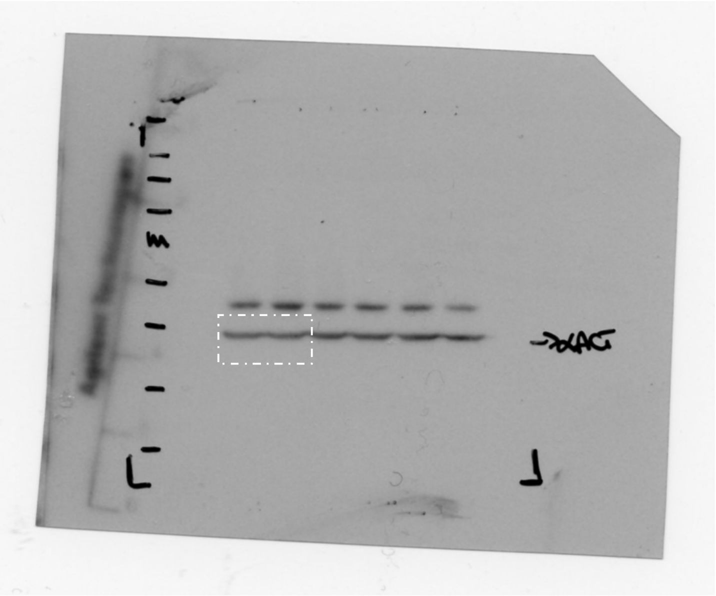

Figure 1E, U373
28S

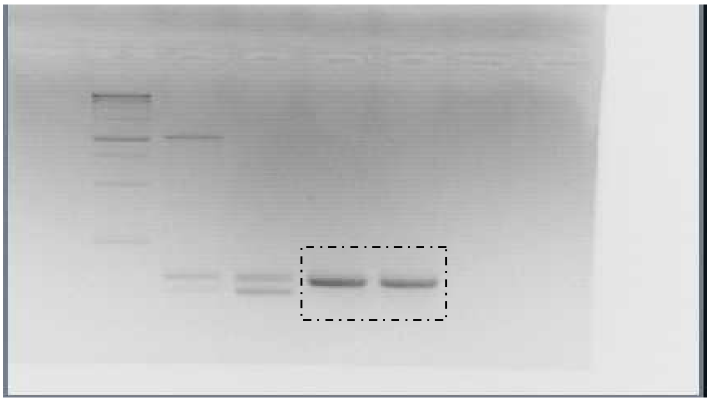

Figure 1F, U373
28S

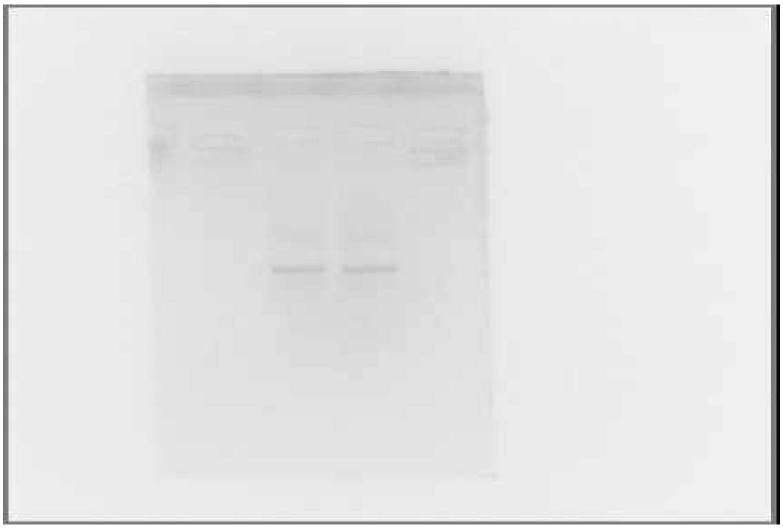

Figure 1F, U373
p53

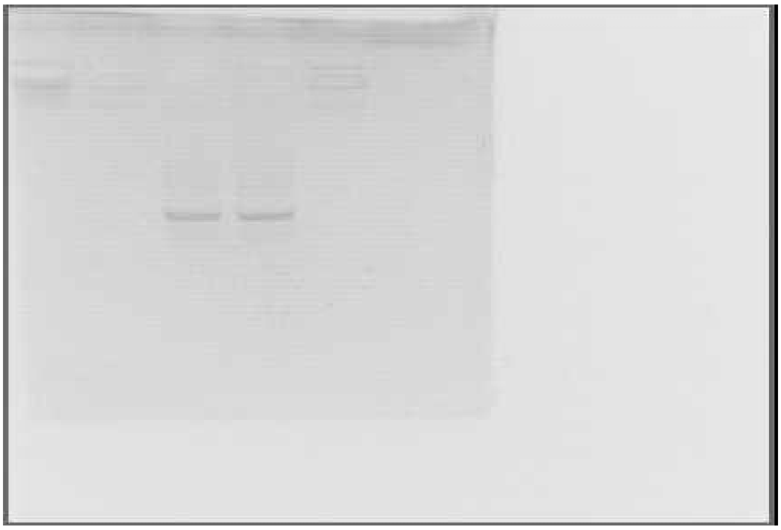

Figure 1F, HT29
p53

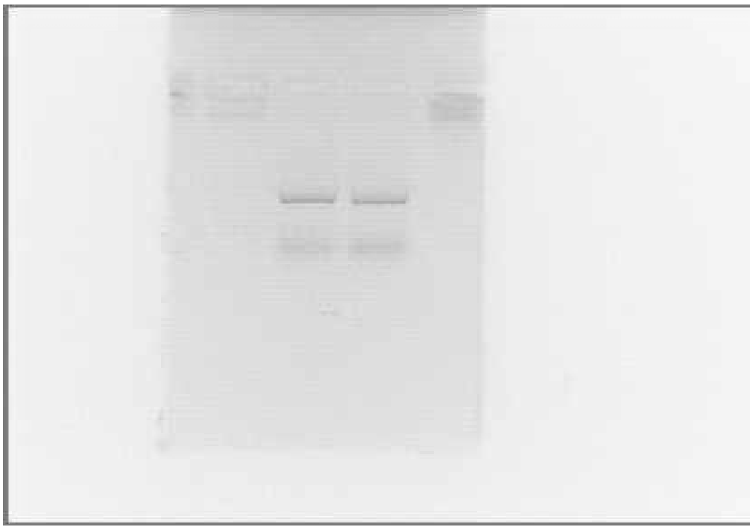

Figure 1F, U373
28S

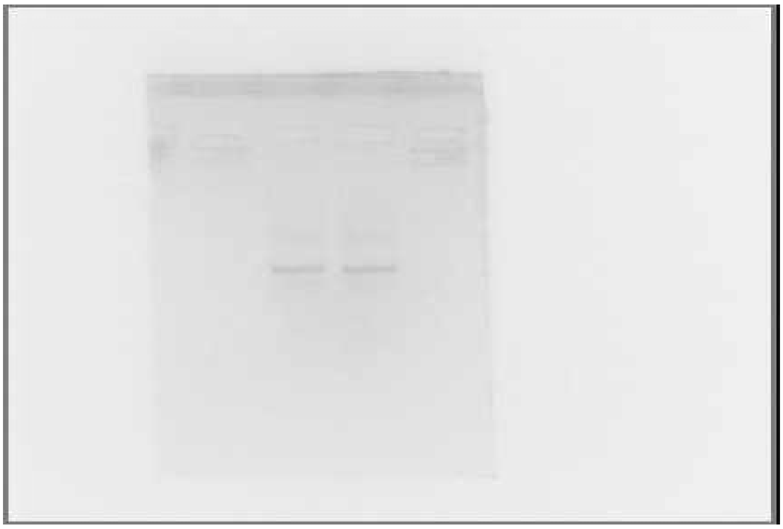

Figure 1F, HT29
28S

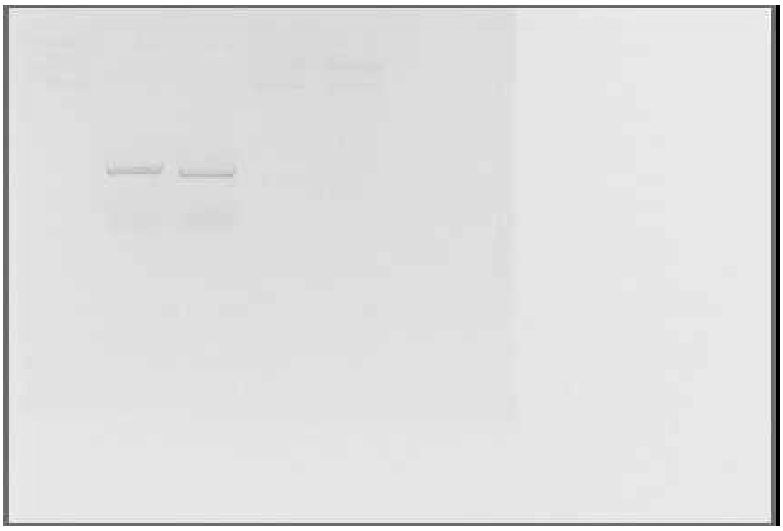

Figure 2B, RKO
actin

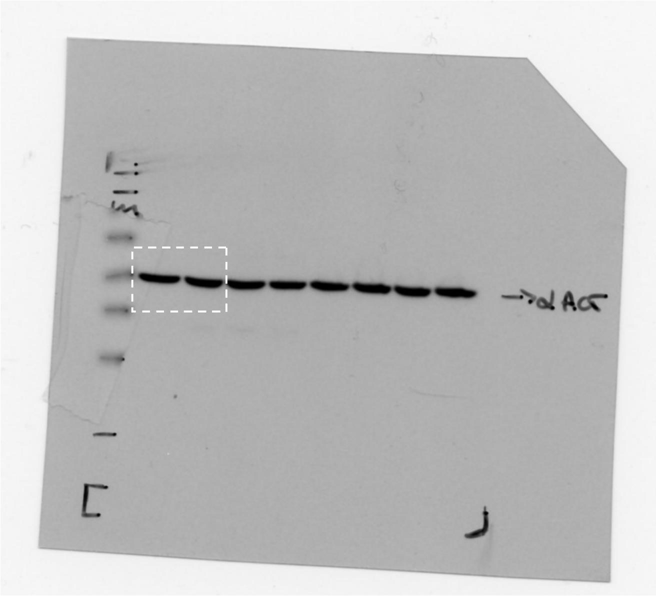

Figure 2B, HCT116
actin

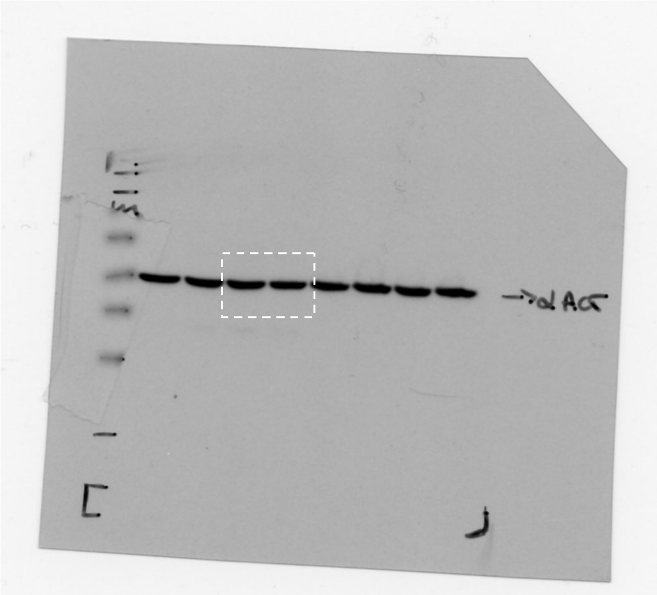

Figure 3C
28S

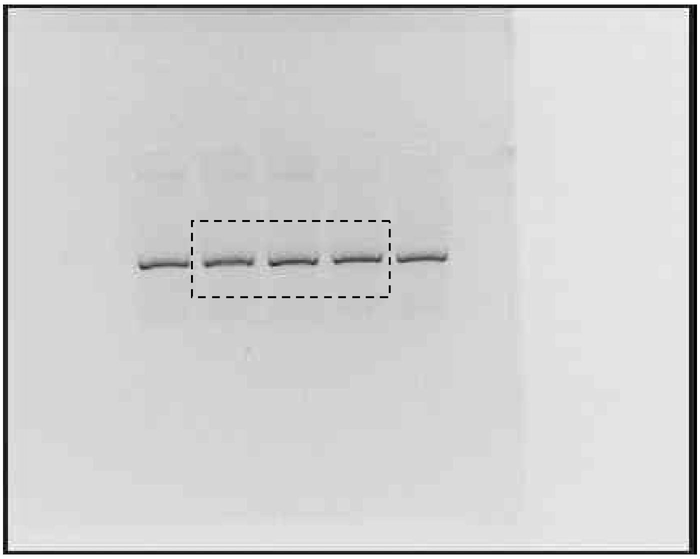

Figure 4A
28S

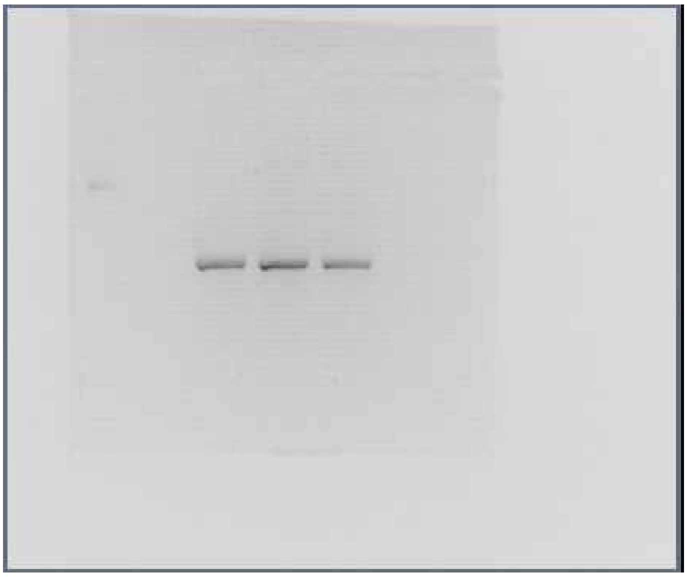

Figure 4B
actin

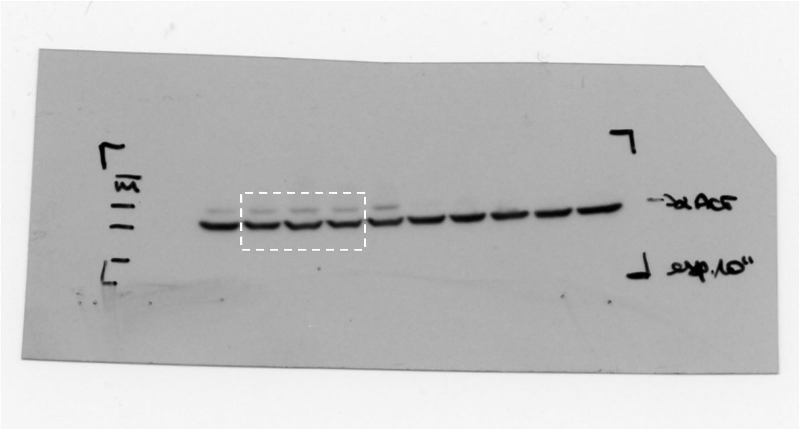

Figure 6B
actin

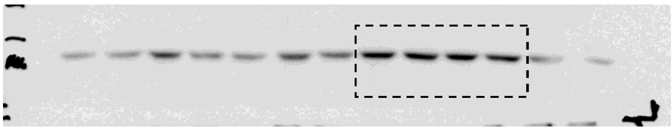

U373

	subG1		
Mock	2		
	2		
Zn	20	22	2,83
	24		
Zn+4-BPA	8	7	1,41
	6		
Zn+STF	9	7,5	2,12
	6		
Zn+PFT	9	7,5	2,12
	6		

HT29

	subG1	
Mock	2	
	2	
Zn	22	23
	24	
Zn+4-BPA	12	10
	8	
Zn+STF	10	8
	6	
Zn+PFT	9	7,5
	6	

1,41

2,83

2,83

2,12
